# A one health approach for integrated vector management monitoring and evaluation

**DOI:** 10.1016/j.onehlt.2024.100954

**Published:** 2024-12-18

**Authors:** Johanna Fite, Thierry Baldet, Antoinette Ludwig, Sylvie Manguin, Claude Saegerman, Frédéric Simard, Philippe Quénel

**Affiliations:** a ANSES, Risk Assessment Department, 14 rue Pierre et Marie-Curie, 94700 Maisons-Alfort, France; b ASTRE, Cirad, INRAE, Univ. Montpellier, Plateforme Technologique CYROI, Sainte-Clotilde, Réunion, France; c National Microbiology Laboratory Branch, Public Health Agency of Canada, 3200 rue Sicotte, C.P. 5000, St. Hyacinthe, QC, Canada; d Research Group on Epidemiology of Zoonoses and Public Health (GREZOSP), Faculty of Veterinary Medicine, Université de Montréal, Saint-Hyacinthe, QC, Canada; e HSM, Univ. Montpellier, CNRS, IRD, Montpellier, France; f Research Unit of Epidemiology and Risk Analysis Applied to Veterinary Science (UREAR-ULiège), Fundamental and Applied Research for Animal and Health (FARAH) Center, University of Liège, 4000 Liège, Belgium; g MIVEGEC, Univ. Montpellier, IRD, CNRS, Montpellier, France; h Univ. Rennes, EHESP, Inserm, IRSET UMR_S 1085, F-3500 Rennes, France

**Keywords:** Arbovirus, Assessment tool, Evaluation, Integrated vector control (IVC), Mosquito, One health, Vector-borne disease

## Abstract

The French Agency for Food, Environmental and Occupational Health & Safety (Anses) has set up a multidisciplinary working group (WG) to develop an innovative One Health approach for the monitoring and evaluation of an integrated vector management system (IVMS) on a territorial scale. Four existing evaluation guidelines and methods have been combined into a semi-quantitative evaluation approach that takes into account all the dimensions of an integrated process. We propose a set of 34 criteria divided into three sections (objectives and management, implementation, integration) that correspond to the main functional components of an IVMS. Each criterion is assigned a score based on the results of a scoring questionnaire completed by the system's stakeholders, and two graphical outputs are generated using a specific combination of these scores. An overview of the system's performance is provided through a series of pie charts synthesizing the scores for each of the three sections and the corresponding eleven subsections. A radar chart further combines the results according to eight attributes chosen to characterize the qualities of the system. Our approach was tested for the invasive mosquito *Aedes albopictus,* a main vector of arboviruses, in two French territories with contrasting dengue epidemiology. This approach is intended to be generic and usable in all territories that are at risk of being affected by arboviruses, whether in tropical or temperate regions. Beyond a conventional assessment of the various components of an IVMS, our interdisciplinary and multisectoral approach aims to gain a better understanding of such a system in its environment, its overall functioning and its mechanisms for adapting to contextual change. It also aims to identify avenues for improvement as part of a continuous quality process, and to facilitate comparisons between territories and the cross-fertilization of knowledge between stakeholders.

## 1 Introduction

The risk of infectious agents transmitted by arthropod vectors is a worldwide public health issue. This is the case for dengue fever, a viral disease transmitted by certain species of *Aedes* mosquitoes, whose incidence has increased dramatically over the past decade [1], with severe epidemics currently affecting Latin America, Asia and the Indian Ocean [[2], [3], [4], [5], [6]]. In 2023, the highest number of dengue cases was reported in the Americas region, with a total of 4,565,911 cases, including 7653 (0.17 %) severe cases and 2340 deaths (case fatality rate of 0.051 %). This situation of high transmission has continued into 2024, in which 673,267 cases of dengue were reported from epidemiological week (EW) 1 to EW 5, including 700 severe cases (0.1 %) and 102 fatal cases (case fatality rate 0.015 %) [7]. Other arboviruses also transmitted by *Aedes* mosquitoes, such as chikungunya, Zika and yellow fever, continue to occur in many parts of the world [[8], [9], [10]], including in temperate regions of the northern hemisphere such as the USA and Europe.

Reunion and Mayotte Islands are two French overseas departments located in the south-western Indian Ocean. Both islands have experienced unprecedented chikungunya epidemics in 2005–2006, with a cumulative incidence rate of 35 % [11]. Moreover, both have experienced dengue outbreaks with more than 30,000 cases reported on these islands between 2017 and 2022 [5,12,13] and dengue now appears to be endemic on Reunion Island [14]. The French West Indies territories have been endemo-epidemic for over two decades for dengue, as illustrated by the recent dengue epidemics in Martinique, Guadeloupe and French Guiana [15]. Mainland France has also been affected by arboviruses ever since the invasive tiger mosquito *Aedes albopictus* became established in 2004 and expanded its range*,* and since 2010, there have been recurrent indigenous cases of dengue fever and chikungunya in the south of France [9,16]. In 2022, the dengue virus (DENV) transmission situation in mainland France was exceptional in terms of both the number of transmission events and the number of autochthonous cases, with an unprecedented peak of 65 cases spread out over 9 transmission events between July and late September [17]. This situation was repeated in the summers of 2023 (45 cases) and 2024 (83 cases), with new local outbreaks in the south of France and, for the first time, respectively indigenous cases of dengue and one of chikungunya occurring near Paris [16,18].

A real public health issue, the arboviral diseases epidemics have a major impact not only on healthcare provision (overflow of healthcare facilities), but also on societal activities in general (e.g., municipal budgets, absenteeism at work and school) [19]. With global changes (in particular, climate, land use, urbanization and increased transport of goods and people), emerging arboviruses represent an increasing risk to human and animal populations [20,21]. Therefore, vector-borne diseases have become a major health and safety issue and the subject of high societal concern, particularly with respect to the acceptability and effectiveness of vector control (VC) methods and strategies [22]. According to the World Health Organization, “Never has the need for a comprehensive approach to vector control to counter the impact of vector-borne diseases been more urgent” [23].

As part of the reform of vector control governance implemented in France in 2019, the French Agency for Food, Environmental and Occupational Health & Safety (Anses) was asked by the French Ministry of Health to evaluate vector management systems in the French territories, including overseas. Besides assessing the various components of an integrated vector management system (IVMS) (i.e., entomological and epidemiological surveillance, vector control, social mobilization and communication), it was expected that this approach would help better align the integrated management strategy with the local environment and territory-specific contexts (i.e. regional administrative level in France or the level at which decisions are taken), in addition to proposing ways to improve the overall functioning of the system and its ability to adapt to contextual changes. Thanks to its standardized design and reproductibility, the approach should also be part of an ongoing quality process and facilitate comparisons between territories in terms of their vector management system design and overall efficiency.

To achieve these objectives, we reviewed existing evaluation tools and developed an ad hoc methodology to jointly assess (i) the objectives and governance of the IVMS, (ii) its implementation, and (iii) interactions between the different components of the system and between stakeholders in a One Health perspective. Indeed, the One Health paradigm is particularly relevant in the context of Integrated Vector Management, which aims to control vector-borne diseases through a multifaceted strategy that incorporates various disciplines and sectors. The integration of these elements is essential for developing effective and sustainable vector control strategies that can adapt to the complexities of disease transmission dynamics.

This paper presents the original methodology used to develop such an approach, known as IVM-Ev (acronym for Integrated Vector Management Evaluation), as well as its final framework [24], which was refined following two pilot studies carried out under real field conditions.

## 2 Material and methods

### 2.1 Work process

A multidisciplinary working group (WG) of six experts (on entomology, epidemiology, vector control, development, and the use of assessment methods) was set up by Anses.

• The WG studied the content and outputs of four existing tools presented in Appendix 1: (i) the joint external evaluation (JEE) tool [25], (ii) the OASIS method [26], (iii) the WHO Framework for a National Vector Control Needs Assessment, and (iv) the Network for Evaluation of One Health (NEOH) [27].
• The WG evaluated the complementarity of the four tools and the added value of combining their processes, methods and outputs.
• A One Health approach and specific tools for evaluating IVMS based on the surveillance and control of *Aedes* mosquitoes, which are vectors of arboviruses in France, were developed (called IVM-Ev).
• This initial version (v0) of the IVM-Ev approach was reviewed by experts in entomological surveillance, vector control and evaluation processes.
• A revised version (v1) was tested during pilot studies carried out under real field conditions in two French territories with very different epidemiological contexts for dengue.
• A final (v2) version integrating the lessons learned from the pilot studies was produced.

### 2.2 Review of existing tools

The analysis of the four methods presented in Appendix 1 showed that nearly all of them follow the same basic process: an assessment team is set up, an on-site evaluation is conducted to collect all data relevant to describe the structure and implementation of the strategy, a questionnaire or a checklist is used to collect these data, the data are analyzed and a statement of conclusions and recommendations is produced. However, each tool had a specific feature that the WG considered particularly well-suited to the target objectives:

1. The JEE-IHR: The WG was inspired by the JEE-IHR technical framework's two-step evaluation, which is based on positive interactions between an internal and an external evaluation team and its principle of cross-collaboration (with actors from territories other than the one being evaluated), which promotes experience sharing;
2. OASIS: The WG was inspired by OASIS’ principle of data collection using a four-level scale scoring questionnaire, the presentation of evaluation results in graph form (pie and radar charts) and the list of attributes depicting system performance;
3. The Framework for a National Vector Control Needs Assessment (VCNA): The WG was inspired by VCNA (particularly by VCNA's Annex 1: Vector Control Needs Assessment Questionnaire) for the formulation of the IVM-Ev question guide;
4. NEOH: The WG was inspired by NEOH's One Health systemic approach, which enables an integrated analysis of the system's organization across all of its components.

These four methods differ significantly in the way information is compiled and processed. While one is semi-quantitative (the OASIS method), the others are based on standardized qualitative assessment criteria (the JEE tool, NEOH and VCNA).

### 2.3 Building of the IVM-Ev

IVM-Ev was conceived as an approach designed to address and evaluate an IVMS implemented at a territorial scale corresponding to the “decentralized” administrative level at which decisions are taken (for example, the departmental or regional level in France) and where governance is implemented for the deployment of prevention and control of infectious diseases, particularly those transmitted by vectors. Moreover, in mainland France and overseas, this administrative level corresponds to a territorial scale with specific geographical, climatic, environmental and socio-economic characteristics. With this in mind, a functional approach was used to describe and analyze all the relevant dimensions of an IVMS, including decision and implementation chains, quality control, data mining, and interactions between stakeholders.

We therefore considered the possibility of developing an information collection questionnaire that would cover most of the useful information and concentrate more on the elements that need to be included in the implementation of an IVMS (decision making, management, integrated surveillance, impacts, etc.).

**Fig. 1:**
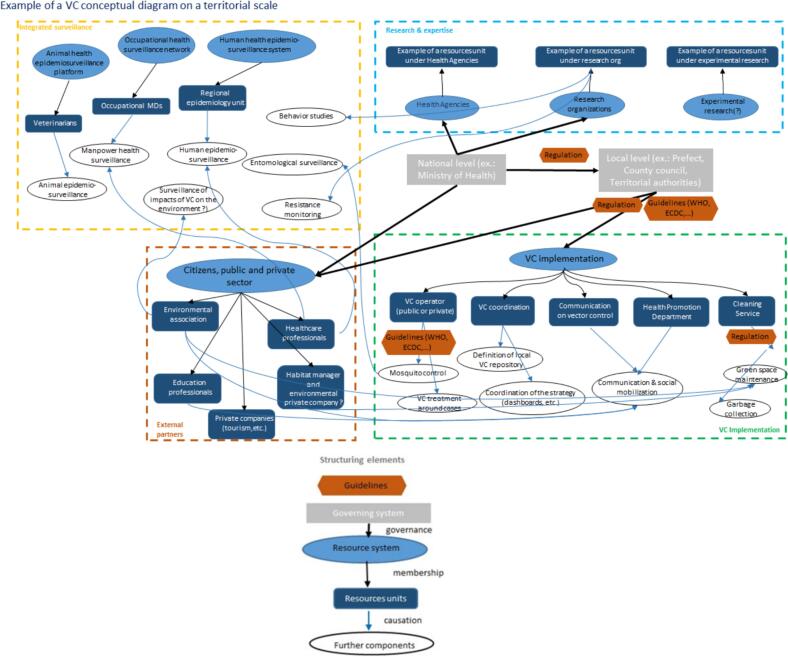
Conceptual diagram of an Integrated Vector Management System, with examples for each category, and list of key structuring elements to consider when building the IVM-Ev questionnaire. The conceptual diagram is intended to help those implementing the IVM-Ev approach to construct a diagram of their own system, identifying the key players and their activities, including the most peripheral ones (upper panel), as well as the key links between players and the framework documents to be taken into account in the assessment. The proposed diagram provides a basis that can be simplified or complexified according to the user's context, while ensuring that a one Health perspective is maintained as far as possible [27].

Fig. 1 provides a conceptual diagram of an IVMS and a list of the key structuring domains and elements to consider when building the IVM-Ev questionnaire.

From this mapping (see Output 1), a questionnaire based on the OASIS tool was developed. The structure of the sections and questions was adapted to conform to the expected structure of an optimal IVM. We developed a questionnaire that strikes a balance between questions that are overly specific, which do not allow us to address all possible components of a vector control strategy, and questions that are too general, which result in imprecise answers. The number and nature of the questions were also adjusted in order to evaluate all the components identified as part of the strategy. Eight attributes characterizing the qualities of the system were then identified and rated with the help of a group discussion aimed at reaching a consensus on the final decision. The questions needed to address the main components of an IVM and provide information on its operation and performance. We therefore developed a set of scoring criteria, which enabled us to obtain a semi-quantitative assessment of all the activities and structures of an IVM, presented as pie charts (see Output 2). Each question was used only once to feed one of the eight attributes, presented as a radar output (see Output 3).

Inevitably, the development of the scoring questionnaire and combination of assessment criteria are subjective in nature. However, we attempted to reduce this subjectivity by applying a consensus process within the WG.

We then submitted a v0 version of the IVM-Ev and related tools to four external experts on assessment methods and/or vector control. After analyzing and discussing their feedback, we tailored a v1 version of the IVM-Ev, clarified some definitions and the objectives of the related tools and modified the items on the scoring questionnaire.

### 2.4 Pilot studies of the IVM-Ev approach in Occitanie and Reunion Island

The v1 version of the IVM-Ev and its associated tools were tested under real conditions for *Aedes albopictus* in order to assess (i) understanding of the approach and its acceptability by stakeholders, (ii) use of the tools by actors, (iii) appropriate use of the questionnaire, (iv) the time required for the various evaluation phases, and (v) overall feasibility (Table 1).

**Table 1:** Pilot studies of the IVM-Ev approach in Occitanie and Reunion Island.

	*Occitanie*	*Reunion*
Number of persons on the internal team	7	6
Number of persons on the external team	7	6
Observer	–	1
Duration of the on-site visit (days)	3	5
Number of meetings with collaborators and/or other stakeholders	6 + 5⁎	19
Total duration⁎⁎ of the IVM-Ev	September 2021–April 2022	March 2023–September 2023

⁎ These stakeholders were consulted by questionnaire by the internal team only, there was no meeting with the external team.

⁎⁎ The IVM-Ev can be performed at any time of the year but it is better to carry out the evaluation outside the vector's period of activity to ensure availability of all local stakeholders.

To ensure the suitability of the approach in all situations, pilot studies were conducted in two French territories with highly contrasted dengue epidemiology: mainland France (Occitanie), where autochtonous cases of dengue have been detected for several years following imported cases [17], and a French overseas territory (Reunion Island, southwest Indian Ocean) where dengue is endemo-epidemic [28].

The external team reviewed the results of each internal evaluation, and on-site visits were organized. In each territory, the internal team presented the results of its auto-evaluation to the external team, which interviewed a panel of stakeholders involved in IVM at the local level, including state and city services (NGOs and private companies). Both the internal and external teams discussed their evaluation through constructive dialogue, agreeing on the final scores and results of the joint evaluation. Tailored recommendations were collectively drawn up and recorded in a final report.

The teams that used the tools felt that their outputs described their system appropriately and that no irrelevant results were found. The teams recognized that the implementation of the IVM-Ev approach compelled the coordination team of the IVMS to review all of its activities and hold further discussions with their partners to identify improvements, which represents a valuable step forward. In addition, the IVM-Ev approach enabled stakeholders to be more involved in the system and to set up exchanges with all those involved in vector control.

The on-site visits and meetings were particularly valued as assets of this approach.

Following the pilot studies, the IVM-Ev tools were reviewed for both content and form. Some questions in the scoring questionnaire were clarified, and the total number of questions was significantly reduced. A scrolling menu was also added to the questionnaire to facilitate scoring.

In conclusion, these two pilot studies validated the process (building of the teams, preparation phase, on-site visits, etc.) and the overall approach.

## 3 Results

### 3.1 Development of a one health approach for integrated vector management monitoring and evaluation (IVM-EV)

#### 3.1.1 Scope of application

The IVM-Ev approach is primarily intended for stakeholders (e.g., regional health agencies, operators, communities) directly involved in the development and management of an integrated vector management strategy on a territorial scale, and all managers and decision-makers interested in the results of the evaluation (e.g., Public Health Agency, Ministry of Health, Ministry of the Environment), as well as experts invited to take part in the external evaluation.

Most vector management systems currently in place worldwide are based on a set of tools and standards (e.g., regulations, WHO and ECDC guidelines) set out in various documents (e.g., plans, job descriptions, quality manual) that constitute the “regional vector management monitoring reference”. The IVM-Ev approach was developed to evaluate the vector control strategy being implemented in a given territory. The approach was first developed for *Aedes* mosquitoes because of their major role in arbovirus transmission, but the approach could be considered for other mosquitoes or even other arthropods.

#### 3.1.2 A two-step evaluation: internal and external evaluation teams working closely together

*Internal phase (self-assessment)*. In this initial stage, the internal evaluation team, made up of actors in the vector management strategy, completes the scoring questionnaire as part of a self-assessment process. The internal evaluation team uses the IVM-Ev tools (questionnaire and spreadsheets) to guide the process. The results and recommendations of any previous evaluation should be incorporated into the self-evaluation. The internal team should identify and reference supporting documents, including legislation, policies, regulations, plans and the results of other assessments. The result is a self-assessment of local vector control actors across the 11 subsections (corresponding to the different technical domains presented in Fig. 3). This document serves as baseline information for the external evaluation and must be submitted to the external evaluation team at least two or three weeks prior to the on-site visit.

*External evaluation phase*. The external evaluation team is made up of multi-sector experts (e.g., entomologists, epidemiologists, sociologists, vector control specialists). The team begins by examining the self-evaluation report and the files of supporting documents and assessments, which are sent prior to the on-site visit. The core of the evaluation mission consists of multi-sectoral and fully collaborative peer-to-peer discussions.

**Fig. 2:**
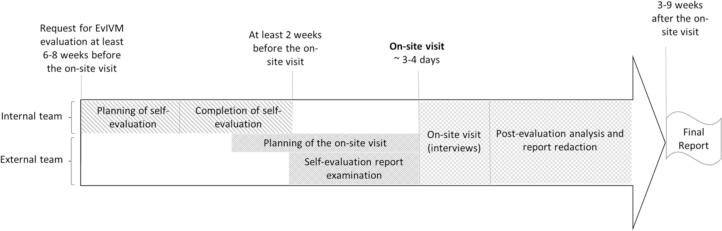
Flow diagram illustrating the progress of an IVM-Ev assessment.

The flow chart for an IVM-Ev assessment is depicted in Fig. 2.

#### 3.1.3 Scoring questionnaire

We consider three main sections in the questionnaire: the first one focuses on the objectives and the vector control management; the second concerns the implementation of the vector control strategy; and the third deals with the integrated dimension of vector management.

A list of 34 questions describing the operation and implementation of the vector management strategy against mosquitoes was developed (Sup Mat 2). These questions are divided into 11 subsections according to the standard structure and activities of integrated vector management.

**Fig. 3:**
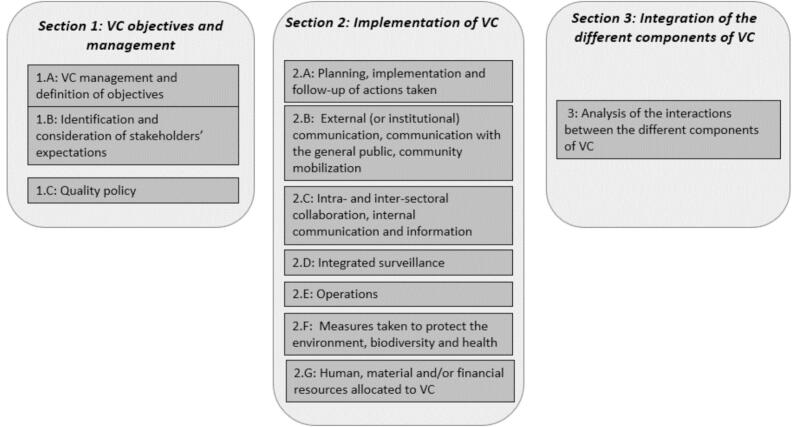
Sections and subsections of the IVM-Ev questionnaire. Note that Section 3 is counted both as a section and a subsection.

The 11 subsections are shown in Fig. 3.

The questionnaire has been designed to collect information useful for scoring the assessment criteria. Multiple criteria are used to summarize a section (from 1 to 24 depending on the section). Each question is scored on a scale of 0 to 3 according to the level of compliance of the system examined. Questions are rated ‘not applicable’ if they are not relevant to the vector control management considered, and this criterion is not taken into account thereafter. Questions are scored using a scrolling menu that describes the conditions that must be met for a given score to be awarded. An example of a scrolling menu for one criterion is given in Table 2.

**Table 2:** Example of a scrolling menu for assessment criteria 1.1.2 ‘*Are the objectives of the vector control strategy in line with the territory's needs?’*

Score	Standard of application (in the scrolling menu)
3	The objectives of the VC strategy are in line with the territory's needs and the target vector. Objectives have been defined for each aspect of the strategy.
2	The objectives of the VC strategy are generally in line with the territory's needs and the target vector. Objectives have been defined for several aspects of the strategy, but minor improvements are needed.
1	The objectives of the VC strategy are not entirely in line with the territory's needs and the target vector. Objectives have been defined for some aspects of the strategy, but major improvements are needed.
0	No objectives have been defined.

Once scoring is completed in the first sheet (column “E”) of the Excel® spreadsheet (Sup Mat 2), each output is generated using a specific combination of the scores and automatically calculated.

#### 3.1.4 Outputs

Three main types of outputs are produced.

#### 3.1.5 Output 1

For a comprehensive portrait of the system's organizational structure, we developed a theoretical conceptual scheme of a vector management system, inspired by the NEOH approach [29]. This scheme helped to visualize all the actors and the complexity of their inter-connections at a local level, and identified the key interactions of such a system for a One Health approach.

As shown in Fig. 1, there are three key steps in the evaluation process when building the conceptual diagram of IVMS at a local level. The first step is to list the key elements to be considered (see the examples in each category in Fig. 1 for descriptions of the context, i.e., the system). The second step is to further define the subsystems by identifying the guidelines and governance system, the resources system and units, and other components for each element under consideration. The third step is to consider the links (Governance, Ownership, Causality) between the different resources (i.e., the structuring elements in Fig. 1).

An example of a conceptual diagram of an IVMS at a local level is given in Appendix 2. This IVMS might vary greatly between territories (in terms of the steering committee, scientific and technical committee, stakeholders, operators, etc.).

#### 3.1.6 Output 2

**Fig. 4:**
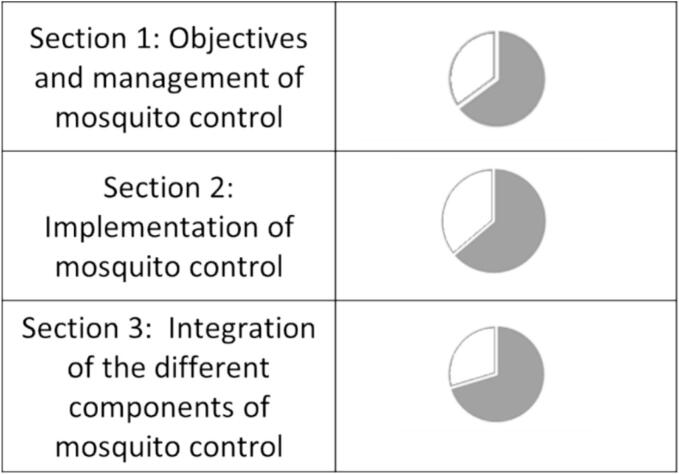
Output 2: Pie chart showing the results of the scores for all criteria in the section. In each pie chart, the gray area represents the percentage of section objectives that have been achieved, and the white area is the percentage of objectives that have not been achieved.

Each section is summarized by a pie chart representing the result of the scores obtained for all the questions in the section (Fig. 4). The assessment criteria's contribution to the section result is not weighted. Output 2 is considered to be an overview of the structure and implementation of the vector control strategy. This series of pie charts is used to identify weaknesses in the system. They are automatically generated in the second sheet of the Excel® spreadsheet (Sup Mat 2).

The example pie charts (Fig. 4) show a percentage (0 % to 100 %) for each section and indicate the extent to which the ideal objectives of a vector control system were achieved for each section.

#### 3.1.7 Output 3

For Output 3, eight attributes were chosen to represent the qualities of the IVMS. The definitions used by the WG for each attribute are as follows:

1. **Effectiveness** describes how well the IVM objectives were achieved. It measures the extent to which the results achieved meet the objectives set at the beginning (hence the importance of having clear objectives from the outset).
2. **Feasibility** determines whether the strategy is feasible, taking into account the context, resources and planned objectives;
3. **Flexibility** is the ability to adapt the system over time and at any given moment. System Resilience Criterion (resilience includes flexibility and sustainability/viability);
4. **Relevance** assesses the extent to which the objectives of the actions correspond to the expectations of the beneficiaries and the needs of the territory (adaptation to the local context);
5. **Internal consistency** assesses coherence between different actions in the strategy and between their impacts on different time scales;
6. **Impact** assesses how the potential impacts and consequences of the vector management strategy (whether positive or negative, planned or unforeseen) can be avoided in the medium and long term;
7. **External consistency** evaluates whether the vector management strategy addresses a need. How many structures are working to meet this need? Do these structures complement or compete with each other? and
8. **Acceptability** analyzes and evaluates the level of approval, support and participation of various stakeholders in the vector control strategy.

Output 3 is automatically generated in the third sheet of the Excel® spreadsheet (Sup Mat 2).

**Fig. 5:**
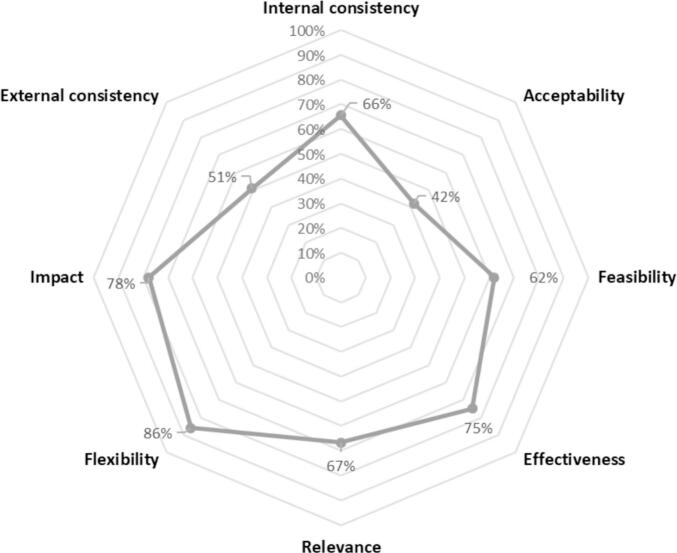
Output 3: System attributes used to assess the quality of an IVMS.

Each attribute is calculated using a specific combination (sum) of the criteria. No weighting was introduced into the calculation. The results of the attribute assessments are plotted on a radar chart to clearly visualize the strengths and weaknesses of the IVMS (Fig. 5).

The radar chart was chosen to easily differentiate Output 3 from Output 2. Each attribute is given a percentage, with 100 % representing optimal quality. However, the percentage could be misinterpreted. For example, a percentage of 86 % for flexibility should not be interpreted to mean that the actual flexibility of the vector control system was estimated using quantitative methods. Rather, given the semi-quantitative approach underlying its calculation, this rate should be understood as indicative of a satisfactory, although sub-optimal ability to adapt to changes.

Meanwhile, in the example illustrated in Fig. 5, Output 3 highlights a clear lack of acceptability and room for improvement in external consistency, while the other attributes appear to be fairly good.

This first sheet of the Excel® spreadsheet (Sup Mat 2), which is the only one to be completed by the users, allows them to include a comment for each score and at the end of each section. Comments can provide additional explanation of the score chosen or a recommendation on how to improve the score.

#### 3.1.8 Duration of a complete IVM-Ev process

Nearly all of the recruitment processes for the evaluation teams took several (six to seven) months to complete (Fig. 2). Although our evaluations were carried out in the context of testing the applicability of the approach, we believe that this duration is representative of the complete evaluation process that would be conducted with the final version. This length of time is comparable to that of other methods (like the JEE tool [25] and the OASIS method [26]), which require a few weeks or even months to ensure the appropriate involvement of the various stakeholder systems and on-site verification, especially when the internal assessors are not involved in the day-to-day activities of the IVMS.

#### 3.1.9 Available resources

The IVM-Ev approach consists of (i) a presentation of the approach, its objectives, principles, and how it should be implemented (Sup Mat 1); and (ii) a data collection and scoring questionnaire implemented in an Excel® spreadsheet for score calculation and visualization of results (Sup Mat 2).

To facilitate the use and improvement of the IVM-Ev, all the necessary resources are available free of charge online (www.anses.fr, in French and English) and presented in the Supplementary Material.

## 4 Discussion and conclusion

### 4.1 A One Health and integrative approach

This work presents the process of developing and implementing a One Health approach for the evaluation and monitoring of IVMS. Evaluating such systems is challenging because of their complexity, which spans a range of activities (e.g., interventions, communication, collaboration) involving a variety of actors and sectors that must work together interactively and synergistically. Moreover, these systems have territory-specific ecological, epidemiological and socio-economical contexts, none of which can be considered independently. Conducting an evaluation in such conditions requires a deep understanding of the dynamics of all system components and their interactions. To deal with this challenge, we have developed a One-Health approach (IVM-Ev) that is innovative in the field of vector-borne diseases and builds on the strengths of existing assessment tools.

### 4.2 A user-friendly tool

The IVM-Ev approach attempts to ease the work of vector management system evaluators by providing them with a questionnaire and a complete scoring process of 34 questions that produces two complementary assessment outputs. The IVM-Ev approach is based on the combined use of a questionnaire spreadsheet and a presentation of the approach to help facilitate the standardized, objective and accurate use of the questionnaire. The structure of the questionnaire is relatively intuitive, and the Excel® spreadsheet is user-friendly. These IVM-Ev tools are very similar to the OASIS tool in its design, which is likely an advantage given that the OASIS tool already has a long lifespan and has been used to perform numerous evaluations of surveillance systems ^in recent years^ [30]^.^ The detailed scrolling menu in the Excel® file appear to be of great help for the evaluation teams, enabling the assessment criteria to be scored unambiguously. The scoring of each criterion and the use of the scrolling menu clearly highlight opportunities for improvement and enable specific recommendations to be made. All of these practical considerations validate the applicability and the ease of use of a single list of questions to generate the various graphical outputs of the system.

For questions that remain difficult to answer due to the lack of available data on specific aspects of the applied vector control strategy, it is recommended that the worst-case scenario and the lowest score be used in the absence of data. Fig. 5 illustrates this situation with an example of a vector control strategy lacking acceptability data.

### 4.3 A semi-quantitative and standardized approach

The IVM-Ev is a semi-quantitative monitoring and evaluation approach that should be used to assess the overall relevance and performance of an Integrated Vector Management System.

Two different graphical layouts were chosen to easily differentiate between the two outputs, reducing the risk of confusion. They clearly illustrate complementary aspects of an IVMS (performance and quality), contributing to a comprehensive evaluation of the IVMS.

Output 2 specifies which part and structure of the system should be targeted for improvement, while Output 3 is useful for understanding the overall quality of a system (e.g., a lack of effectiveness clearly highlights a problem).

Output 3 (the radar chart in Fig. 5) appears to complement Output 2 (the pie chart in Fig. 4) and gives a clear interpretation of IVMS qualities.

Although the output figures present percentages, this does reflect that the IVM-Ev approach is a quantitative method. The percentages should be considered as a relative comparison of system characteristics and attributes, not as a comparison between different vector control systems.

At this stage of development, the approach focuses on the territorial relevance and effectiveness of the vector control strategy and its implementation by the stakeholders, without taking into account the economic dimension and cost-effectiveness aspects of the various IVMS options. In today's economic context, where resources are increasingly constrained or limited, this is a dimension that should be considered.

### 4.4 A co-constructive approach

In addition to its systemic approach, referred to as One Health, the IVM-Ev approach has the advantage of fostering discussions among all actors and stakeholders within the system, as well as with the external evaluation team.

The IVM-Ev approach is not intended to support an audit or inspection of the various authorities contributing to an IVMS; it is based on the principle that the internal evaluation team seeks to obtain valid and valuable results for the system that it is evaluating.

These rich discussions are supplemented by meetings (6 to 19 meetings were held in the pilot studies) with collaborators and/or other stakeholders (e.g., municipalities, associations), enabling the evaluation team to gain a better understanding of the vector management system. A panel of identified stakeholders may be invited to attend the presentation of the evaluation findings and to facilitate discussions on strengths/best practices, areas for improvement/challenges, scores and the identification of one to three key priority actions for each subsection that can be taken to improve the system. An executive summary of the evaluation, including high-level recommendations, the scores and one to three priority actions for each subsection, is prepared by the evaluation team and presented to stakeholders at the end for discussion and validation. A draft final report is provided, typically within two to three weeks after the end of the evaluation, and approved by the vector control system managers.

All of these elements contribute to establishing a sound basis for the co-construction of internal and external solutions when dysfunctions, bottlenecks or shortcomings are identified and areas for improvement have been clearly identified. This approach, which is based on the co-development of priority recommendations, can serve as the framework for an action plan for all stakeholders and foster stakeholder commitment.

Open dialogue and active listening among the different actors in the system is key to securing stakeholder commitment and ensuring the success of any public health strategy, including in integrated vector management.

The pilot studies demonstrated that these aspects of the approach were highly valued by local actors.

### 4.5 Experience sharing

The formation of an evaluation team encourages cross-evaluations between different territories. The participation of IVMS actors from territories other than the one being evaluated on the external evaluation team facilitates the sharing of experiences and good practices between IVM actors.

An external actor (from another territory, another Regional Health Agency or an external operator, for example) can also take part in the evaluation or attend as an observer in order to take a critical look at the IVMS in their territory and consider whether future evaluation is needed.

The approach can also be used in the long term, making it possible to compare and improve the performance of one IVMS across time) or of several IVMS concurrently.

### 4.6 An approach adaptable to different contexts and vectors

The IVM-Ev approach was applied to two mosquito management systems located in two different climatic zones, one in a temperate territory (Occitanie, southern France) and the other in a tropical territory (Reunion Island). The IVM-Ev approach is considered relatively easy to implement and very likely to be applicable to a wide range of vector management systems. With a few adaptations, it could be applied to other arthropod vectors.

Although we focused on *Aedes* mosquitoes and the prevention and control of *Aedes*-borne diseases, users can, if necessary, adapt the approach to take into account other mosquito species (e.g., *Anopheles* vectors of malaria parasites, *Culex* vectors of zoonotic arboviruses such as West Nile virus or Usutu virus) or even other arthropod vectors, including vectors of pathogens responsible for zoonoses or animal diseases. Nevertheless, when a territory has to cope with several vector-borne diseases, the IVM-Ev only addresses one vector control strategy at a time. It can be used as often as necessary to evaluate different vector control strategies.

### 4.7 Perspectives

The choice of criteria, the combination of the criteria and possible weightings, as well as the scoring spreadsheets used to produce the various outputs, could be further refined by applying the IVM-Ev approach to other, more diversified vector management systems. The experience gained from applying the questionnaire could be used to refine the spreadsheets in the future.

Further development of the IVM-Ev tools (questionnaire and spreadsheets) could, as a first step, provide a system for quantifying the cost of proposed improvements, allowing the cost-benefit ratio of any improvement to be simulated. The cost-benefit analysis of the improvement could then be simulated before it is implemented.

Finally, in order to be implemented, the IVM-Ev approach requires dedicated human and financial resources. For this reason, our approach requires strong political support on both the local and national levels.

## CRediT authorship contribution statement

**Johanna Fite:** Validation, Project administration, Conceptualization. **Thierry Baldet:** Writing – original draft, Validation, Conceptualization. **Antoinette Ludwig:** Writing – original draft, Validation, Conceptualization. **Sylvie Manguin:** Writing – original draft, Validation, Conceptualization. **Claude Saegerman:** Writing – original draft, Validation, Conceptualization. **Frédéric Simard:** Writing – original draft, Validation, Conceptualization. **Philippe Quénel:** Writing – original draft, Validation, Conceptualization.

## Declaration of competing interest

This article is the result of a working group established by the French Agency for Food, Environmental and Occupational Health & Safety (Anses), which is a public administrative body reporting to the Ministries of Health, the Environment, Agriculture, Labour and Consumer Affairs.

The authors declare that the research was conducted in the absence of any commercial or financial relationships that could be construed as a potential conflict of interest.

## Figures and Tables

**Fig. 6:**
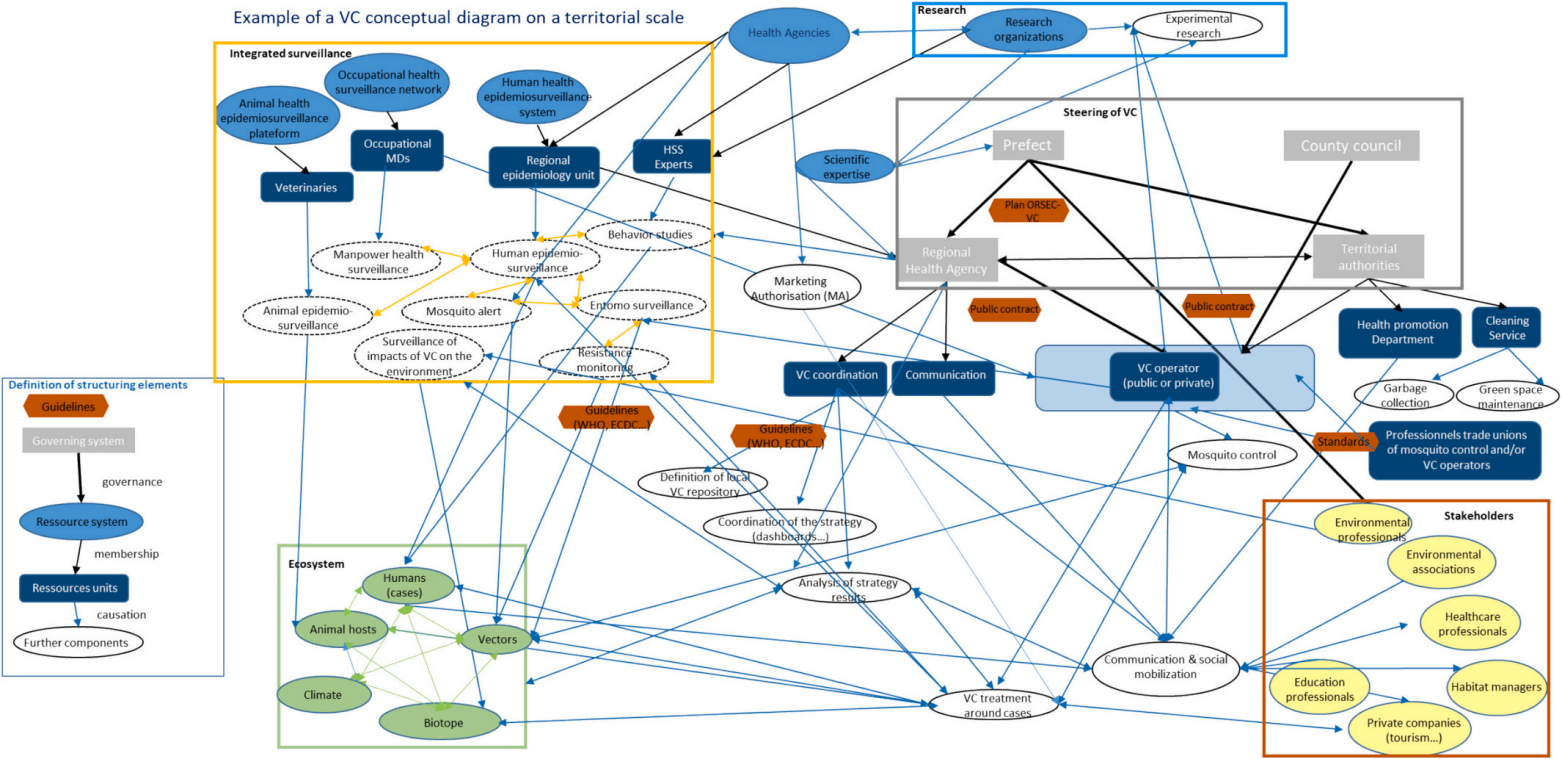
Example of a Conceptual Diagram of an Integrated Vector Management System at a Local Level

## Data Availability

Data will be made available on request.

## Glossary

Anses: the French Agency for Food, Environmental and Occupational Health & Safety.
COST: Cooperation in Science and Technology.
ECDC: European Center for Disease Prevention and Control.
EvLAV: acronym for the French translation of ‘Evaluation of Integrated Vector Management approach.
EW: Epidemiological week.
IHR: International Health Regulations.
IVC: Integrated Vector Control.
IVM-Ev: Evaluation of Integrated Vector Management approach.
IVMS: Integrated Vector Management System.
JEE: Joint external evaluation.
NEOH: Network for Evaluation of One Health.
OASIS: acronym for the French translation of ‘analysis tool for surveillance systems’.
OH: One Health.
VC: Vector control.
VCNA: Vector Control Needs Assessment.
WG: working group.
WHO: World Health Organization

## Appendix A Review of Existing Tools

Four current and complementary assessment tools were used as the basis for developing the IVM-Ev approach: (i) the joint external evaluation (JEE) tool [25], (ii) the OASIS method [26], (iii) the WHO Framework for a National Vector Control Needs Assessment, and (iv) the Network for Evaluation of One Health (NEOH) [27].

### A.1 Joint external evaluation tool (JEE): An International Health Regulations (IHR) application tool

On the basis of the recommendations by the *International Health Regulations* [31] review committee, the WHO has developed a joint external evaluation (JEE) tool [25]. The purpose of this tool is to measure country-specific status and progress towards achieving the IHR targets.

This allows countries to identify the most urgent needs within their health security system; prioritize opportunities for improving preparedness, operational readiness, response and action; and engage with current and prospective donors as well as partners, including local and international non-governmental organizations, to target resources effectively.

The first stage of the process is a self-evaluation using the JEE tool and the country implementation guide. This information is then passed on to the JEE team of international experts [32]. Review of this self-evaluation data enables team members to understand the country's baseline health security capabilities.

The JEE external team uses the same tool for its independent evaluation and works alongside the country team in interactive sessions. After conducting the evaluation, the JEE team drafts a report that identifies status levels for each indicator and presents an analysis of the country's capacities, gaps, opportunities and challenges. The draft report is then shared with the host country. This approach facilitates international support for the country's implementation efforts, encourages the sharing of best practices and lessons learned, promotes international accountability, engages stakeholders, and informs and guides IHR implementation both in the host country and internationally.

### A.2 OASIS: An assessment tool for epidemiological surveillance systems in animal health and food safety

The OASIS (acronym for the French translation of ‘analysis tool for surveillance systems’) is a standardized tool for the assessment of animal health (including zoonoses) and food safety surveillance systems (Hendrikx et al. 2011).

OASIS aims to facilitate the work of surveillance systems evaluators by providing a questionnaire divided into ten sections, representing the functional parts of a surveillance system, and a complete scoring process of 78 criteria that generates three complementary assessment outputs. Each question is scored according to the prescription of a scoring guide. Three graphical assessment outputs can be generated using a specific combination of the scores. Output 1 provides an overview of the system through a series of pie charts synthesizing the scores of each section. Output 2 is a histogram representing the quality of eight critical control points. Output 3 is a radar chart representing the performance of the surveillance through the level achieved by ten system attributes such as (i) simplicity, (ii) flexibility, (iii) data quality, (iv) acceptability, (v) sensitivity, (vi) positive predictive value, (vii) representativeness, (viii) timeliness, (ix) stability and (x) usefulness.

The OASIS package comprises a questionnaire, a list of assessment criteria, a scoring guide, and a spreadsheet for integrating the scoring and generating the outputs.

### A.3 Framework for a National Vector Control Needs Assessment

The Framework for a National Vector Control Needs Assessment [33] has been developed to help stakeholders adopt the Global Vector Control Response - GVCR (2017–2030) guidance [23] for more effective and sustainable vector management. This framework provides a practical tool to help national vector control programs better understand their situation, document their needs for baseline assessment and progress tracking, and mobilize domestic or external resources to address identified needs.

The primary objectives of this assessment framework are to help countries assess the current situation of vector-borne diseases in their country and neighboring areas, and program needs (e.g., policy, structural and infrastructural) to align their vector control program with the GVCR. The framework targets national programs and proposes a step-by-step assessment procedure and a detailed methodology that can be used to conduct a baseline assessment of the situation and needs and to track progress. The framework's standardized structure and approach can also facilitate the comparison of results between countries.

### A.4 NEOH: Network for Evaluation of One Health

The Network for Evaluation of One Health (NEOH), funded by the European Cooperation in Science and Technology (COST), is an open network that brings together experts, researchers and policy-makers from diverse backgrounds interested in the topic [29]. NEOH aims to generate a standardized evidence-based framework on the added value of addressing zoonotic diseases from a One Health perspective. This framework would motivate and encourage enablers and value-chain actors to apply methods following this approach at the relevant scale [27].

By systematically describing the various aspects of OH (One Health), NEOH provides a basis for measuring and monitoring the integration of disciplines, sectors and stakeholders in health initiatives. The framework identifies the social, economic and environmental drivers that result in integrated approaches to health and illustrates how they embody the characteristics of OH operations, i.e., thinking, planning and working, and how they require supporting infrastructures and resources to enable learning, sharing and systemic organization. It also describes the OH outcomes (i.e., sustainability, health and welfare, inter-species equity and stewardship, effectiveness and efficiency), which cannot be achieved through sectoral approaches alone, and how they align with social, environmental and economic-based aspects of sustainable development [34].

## Appendix B Example of a conceptual diagram

This conceptual diagram was developed to describe the Integrated Vector Management System in Reunion Island during the pilot study.

Development of this map was based on a map of the vector control strategy. Mapping was carried out in groups and required several interactions to ensure that all the essential components were captured, including the links between them. The construction of the system map was based on the system map recommended in the NEOH approach [27].

## Appendix C Supplementary data

Excel® spreadsheet with scoring questionnaire: Once scoring is completed in the first sheet (column “E”), each output is generated using a specific combination of the scores and automatically calculated. Image 1
